# CCKAR is a biomarker for prognosis and asynchronous brain metastasis of non-small cell lung cancer

**DOI:** 10.3389/fonc.2022.1098728

**Published:** 2023-01-17

**Authors:** Nan Liang, Suohui Sun, Zheng Li, Tao Wu, Chunpu Zhang, Tao Xin

**Affiliations:** ^1^ Department of Neurosurgery, the Second Affiliated Hospital of Shandong First Medical University, Jinan, China; ^2^ Department of Neurosurgery, the First Affiliated Hospital of Shandong First Medical University and Shandong Provincial Qianfoshan Hospital, Jinan, China

**Keywords:** CCKAR, brain metastasis, non-small cell lung cancer, prognosis, Biomarker

## Abstract

**Background:**

Non-small cell lung cancer (NSCLC) is the most common histological type of lung cancer, and brain metastasis (BM) is the most lethal complication of NSCLC. The predictive biomarkers and risk factors of asynchronous BM are still unknown.

**Materials and methods:**

A total of 203 patients with NSCLC were enrolled into our cohort and followed up. The clinicopathological factors such as tumor size, T stage, lymphatic invasion, metastasis and asynchronous BM were investigated. CCKAR expression in NSCLC and resected BM was assessed by IHC, and CCKAR mRNAs in NSCLC and para-tumor tissues were estimated by qRT-PCR. The correlations between CCKAR expression, BM and other clinicopathological factors were assessed by chi-square test, and prognostic significance of CCKAR was estimated by univariate and multivariate analyses.

**Results:**

CCKAR was highly expressed in NSCLC tissues compared with para-tumor tissues. CCKAR expression in NSCLC was significantly associated with asynchronous BM. The BM percentages for NSCLC patients with low and high CCKAR were surprisingly 5.2% and 66.6%, respectively. CCKAR expression and BM were unfavorable factors predicting unfavorable outcome of NSCLC. Moreover, CCKAR expression in NSCLC was an independent risk factor of asynchronous BM.

**Conclusions:**

CCKAR is a prognostic biomarker of NSCLC. CCKAR expression in NSCLC is positively associated with asynchronous BM, and is a risk factor of asynchronous BM from NSCLC.

## Introduction

Lung cancer is the most common cause of cancer death worldwide, resulting in approximate 1.6 million deaths each year (1). Histologically, lung cancer is mainly divided into small cell lung cancer (SCLC) and non-small cell lung cancer (NSCLC) (2). NSCLC accounts for about 85% of all the lung cancers (3). Brain metastasis (BM) is the most common and lethal event in patients with lung cancer (4). The development of BM remains one of the main factors associated with poor prognosis and mortality in patients with lung cancer. About 150,000 patients with cancer develop into BM in USA each year (5), and lung is the most common primary site for BM (6, 7). Almost 10% of newly diagnosed patients with NSCLC develop metastasis and 25%–40% acquire BM during the course of disease (8). Although multimodal treatments and advances have been used in BM treatment, the prognosis of BM is extremely poor, ranging from 1.5 to 9.5 months (9, 10). More unfortunately, there is no way which can predict NSCLC patients with high risk to suffer BM during the disease course to date.

Cholecystokinin (CCK) receptor, including CCKAR and CCKBR, is a receptor for cholecystokinin, mainly regulating pancreatic growth and enzyme secretion, smooth muscle contraction of the gallbladder and stomach (11). Previous studies mainly suggest that CCKAR is primarily expressed in the alimentary tract, while CCKBR is mainly found in the brain and the stomach (12). Emerging evidence shows that CCKAR is also expressed in central nervous system and modulates feeding and dopamine-induced behavior (13). The role of CCKAR in cancer is also gradually revealed in these years, including hepatocellular carcinoma, gallbladder carcinoma, gastric cancer, et al (14–16). Moreover, targeting CCK receptor is becoming a promising therapy for cancer treatment. However, the role of CCKAR in lung cancer and its metastasis is still unknown.

## Materials and methods

### Patient cohort and ethics

Our study enrolled 434 patients who underwent surgical resection of NSCLC, which comprise of the testing cohort, from 2017 to 2020 in the First Affiliated Hospital of Shandong First Medical University and the Second Affiliated Hospital of Shandong First Medical University. From the testing cohort, a total of 203 patients were selected and their outcomes were followed up. The inclusion standards contain that (i) available formalin-fixed tumor tissues and follow-ups, (ii) no pre-operational adjuvant therapy including chemotherapy or radiotherapy. Patients who died during the perioperative period were excluded from the study. All patients received the standard treatment strategies of NSCLC after lung cancer resection including chemotherapy if tumors were invasive, and target therapy if there existed available mutations (17). The study was approved by the Ethics Committee of the First Affiliated Hospital of Shandong First Medical University, and the Second Affiliated Hospital of Shandong First Medical University (Approval No. 2021-091).

### Immunohistochemistry and evaluation

CCKAR expression in NSCLC and BM was detected and semi-qualified with IHC by streptavidin peroxidase complex method as previous described (18). Slides were treated with boiled citrate buffer (pH = 6.0) for antigen retrieval. The inactivity of endogenous peroxidase was completed by 3% hydrogen peroxide, and unspecific antigen binding was blocked by 5% bovine serum albumin. The primary antibody of CCKAR (1:100, Santa Cruz Biotechnology, Santa Cruz, CA, USA) or CCK-8 (1:100, Abcam, ab27441, UK) was used to incubate the specimens overnight at 4°, and the corresponding secondary antibody labelled with streptavidin-biotin-peroxidase reagent (Beyotime, Beijing, China) was administrated to incubate the specimens for 30 minutes at room temperature. At last, the antigens were visualized by incubation in the 3,3’-diaminobenzidine solution.

The IHC results were semi-quantified by two aspects, the staining intensity and the positive stained cell percentage, which were the IHC score. The IHC score was assessed by two senior pathologists who were unaware of the clinical data. The IHC score was defined as the score of the staining intensity multiplied by the score of positive stained cell percentage. The scores of staining intensity were defined as: score 0, 1, 2 and 3 for negative staining, weak staining, moderate staining and strong staining, respectively. The scores of positively stained cell percentage were defined as: score 1, 2, 3 and 4 for <25% positive cells, 25%-50% positive cells, 50%-75% positive cells and 75%-100% positive cells respectively. The final IHC score varied from 0 to 12. The cohort was divided into subsets with different CCKAR expression by the cut-off of IHC score, which was identified by receiver operating characteristic (ROC) curve.

### RNA extraction and qRT-PCR

qRT-PCR was performed to assess CCKAR expression in fresh NSCLC tissues and para-tumor tissues. The total RNA was extracted by TRIzol agent (Thermo Fisher, Waltham, MA, USA). Reverse transcription of cDNA was accomplished with ReverTra kit (TOYOBO, Japan), and the quantitative real-time polymerase chain reaction (qRT-PCR) was applied with Thermo Fisher 7500 PCR System and SYBR Premix. The quantification of qRT-PCR results was analyzed by the 2^-ΔΔCt^ method with GAPDH as an internal control. The primers of CCKAR and GAPDH were designed as follows: CCKAR, forward: 5’-ATGGATGTGGTTGACAGCCTT-3’, reverse: 5’-AAGCGTCTCATTTTCGAGCCC-3’; GAPDH: forward: 5’-GAGTCAACGGATTTGGTCGT-3’, reverse: 5’-GACAAGCTTCCCGTTCTCAG-3’.

### Statistical analysis

SPSS 22.0 software (SPSS, Chicago, IL, USA) was used to analyze all the statistical significance. The correlations between CCKAR, BM and other clinicopathological factors were assessed by the chi-square test. The statistical difference between subgroups was assessed by the log-rank test, and overall survival(OS) curves were plotted by Kaplan-Meier method. The Cox-regression hazard model was applied to identify the independent prognostic factors. *P* value less than 0.05 was regarded as statistically significant.

## Results

### Expression of CCKAR in primary NSCLC

**Figure 1:**
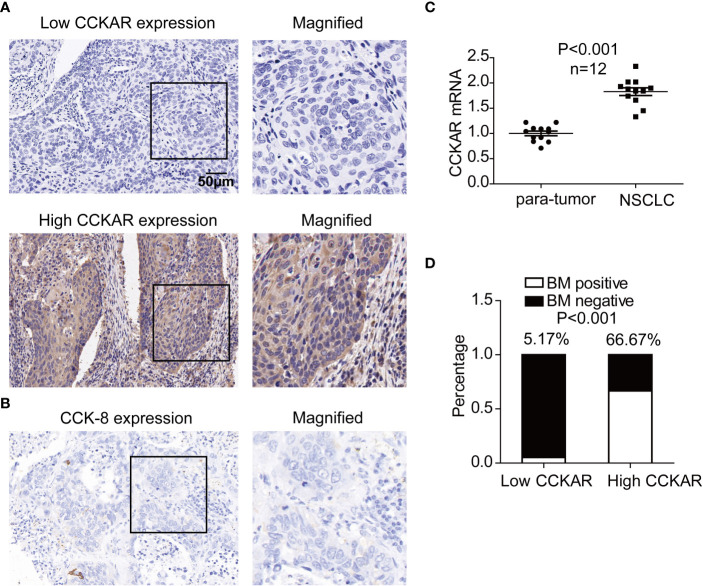
Expression of CCKAR in NSCLC. **(A)** The expression of CCKAR in NSCLC was detected by IHC. Patients with NSCLC were divided into subsets with low or high CCKAR expression. **(B)** Expression of CCK-8 in NSCLC was detected with IHC and representative images were shown. **(C)** CCKAR expression in 12 pairs of NSCLC and para-tumor tissues was detected with qRT-PCR. The statistical significance was evaluated by paired t test. **(D)** Asynchronous BM percentages of patients with low and high CCKAR were calculated and compared with chi-square method. Patients with high CCKAR in NSCLC primary tumor were much more susceptible to BM.

The expressions of CCKAR in NSCLC and corresponding tumor-adjacent tissues were detected with IHC and qRT-PCR. In 203 cases of NSCLC, IHC was performed to show the expression and localization of CCKAR. In our study, CCKAR was mainly expressed in cytoplasm and membrane (**Figure 1A**). The patients were further divided into subgroups with low or high expression of CCKAR, accounting for 116 and 87 respectively. CCK-8 expression was also detected with IHC in NSCLC. In NSCLC, CCK-8 was hardly detected (**Figure 1B**).

In 12 pairs of NSCLC tissues and para-tumor tissues, CCKAR expression was detected with qRT-PCR. As the result, CCKAR in NSCLC was significantly higher than that in para-tumor tissues (**Figure 1C**), suggesting that CCKAR may play an important role in NSCLC progression.

### Correlation between CCKAR, BM and clinicopathological factors

These 203 patients were followed-up and a total of 64 patients suffered from asynchronous BM. The correlations between CCKAR and clinicopathological factors were analyzed with chi-square test (**Table 1**). Interestingly, high CCKAR in primary NSCLC was significantly associated with positive asynchronous BM (*P*<0.001). The number of patients with BM was 58 in high-CCKAR patients but only 6 in low-CCKAR patients. The BM percentages for NSCLC patients with low and high CCKAR were surprisingly 5.2% and 66.6%, respectively (**Figure 1D**). Moreover, CCKAR tended to be significantly associated with advanced TNM stage (*P*=0.087) and female gender (*P*=0.067).

**Table 1:** The correlation between CCKAR expression and clinicopathological factors in NSCLC.

Factors		CCKAR		
	n	Low	High	P*
Sex				
Male	111	57	54	0.067
Female	92	59	33	
Age				
<60	75	46	29	0.356
≥60	128	70	58	
Tumor diameter				
≤3.5cm	95	59	36	0.18
>3.5cm	108	57	51	
Histological grade				
I	121	70	51	0.804
II-III	82	46	36	
T stage				
I+II	130	78	52	0.272
II+IV	73	38	35	
Lymphatic invasion				
Negative	100	63	37	0.097
Positive	103	53	50	
Metastasis				
No	191	109	82	0.932
Yes	12	7	5	
BM				
No	139	110	29	<0.001
Yes	64	6	58	
TNM stage				
I+II	79	51	28	0.087
II+IV	124	65	59	
Chemotherapy				
No	59	34	25	0.939
Yes	144	82	62	
Target therapy				
No	168	94	74	0.453
Yes	35	22	13	

*calculated by chi-square test.

The correlation between asynchronous BM and clinicopathological factors was also analyzed (**Table 2**). Patients with positive lymphatic invasion were more likely to suffer from asynchronous BM (*P*=0.010). More interestingly, target therapy of NSCLC was associated with low probability of BM (*P*=0.044).

**Table 2:** The prognostic significance of CCKAR, BM and other clinicopathological factors.

Factors	Univariate analysis		Multiriate analysis		
	5-year OS rate	P*	HR	95% CI	P^#^
Sex					
Male	21.2	0.258			
Female	24.4				
Age					
<60	27.2	0.233			
≥60	19.6				
Tumor size					
≤3.5cm	33.8	0.006	1		
>3.5cm	12.1		1.36	0.95-1.94	0.095
Histological grade					
I	26	0.173			
II-III	18				
T stage					
I+II	27.6	0.025	1		
II+IV	13.3		0.92	0.64-1.33	0.668
Lymphatic invasion					
Negative	37.1	<0.001	1		
Positive	8.6		2.08	1.46-2.97	<0.001
Metastasis					
No	23.6	0.445			
Yes	8.3				
BM					
No	28.2	<0.001	1		
Yes	9.5		1.41	0.91-2.19	0.123
TNM stage					
I+II	46.7	<0.001			
II+IV	8.8				
CCKAR					
Low	27.8	0.016	1		
High	15.7		1.15	0.75-1.77	0.516
Chemotherapy					
Yes	23.0	0.792			
No	21.2				
Target therapy					
Yes	24.1	0.600			
No	22.4				

*calculated by log-rank test;^#^ calculated by Cox-regression model.

### Prognostic value of CCKAR in NSCLC

**Figure 2:**
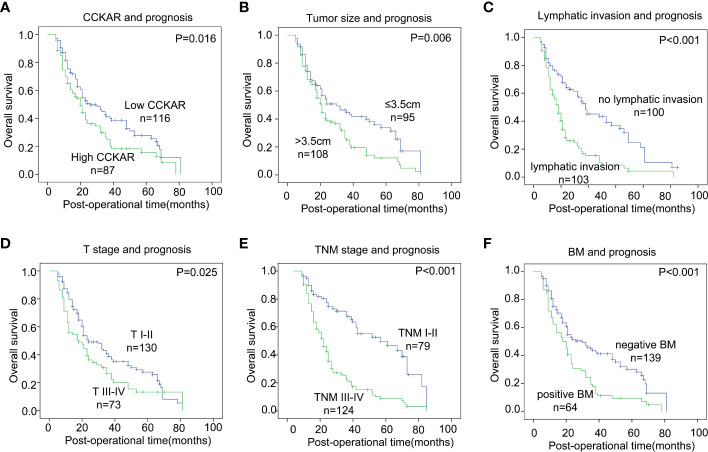
The correlation between NSCLC clinicopathological factors and outcome. NSCLC patients were divided into different subgroups according to CCKAR expression **(A)**, tumor size **(B)**, lymphatic invasion **(C)**, T stage **(D)**, TNM stage **(E)** and BM **(F)**. The statistical significance was calculated by the log-rank test.

The prognostic significance of CCKAR and other clinicopathological parameters were analyzed by univariate and multivariate analyses. The univariate analysis was performed with the log-rank test (**Table 3**). In the univariate analysis, CCKAR was a significant prognostic factor of NSCLC (**Figure 2A**). In addition, large tumor size(*P* =0.006), lymphatic invasion (*P*<0.001), advanced T stage (*P*=0.025) and TNM stage (*P*<0.001) were all substantially associated with unfavorable outcome (**Figures 2B–E**). Moreover, positive BM was a prognostic indicator of poor prognosis (**Figure 2E**).

**Table 3:** The correlation between BM and clinicopathological factors in NSCLC.

Factors	BM		
	No	Yes	P*
Sex			
Male	69	35	0.504
Female	70	29	
Age			
<60	57	18	0.077
≥60	82	46	
Tumor diameter			
≤3.5cm	55	24	0.779
>3.5cm	84	40	
Histological grade			
I	88	33	0.113
II-III	51	31	
T stage			
I+II	93	37	0.212
II+IV	46	27	
Lymphatic invasion			
Negative	77	23	0.010
Positive	62	41	
Metastasis			
No	132	59	0.436
Yes	7	5	
Chemotherapy			
No	37	22	0.258
Yes	102	42	
Target therapy			
No	110	58	0.044
Yes	29	6	

*calculated by chi-square test.

The independent prognostic factors of NSCLC were further validated by multivariate analysis (**Table 3**). The prognostic factors in univariate analysis were enrolled into the Cox-regression model including tumor size, T stage, lymphatic invasion, BM and CCKAR expression. Positive lymphatic invasion was identified as an independent prognostic parameter of NSCLC. CCKAR and BM were not an independent prognostic biomarker of NSCLC mainly because of they had significant correlations.

### Clinical significance of CCKAR expression in BM lesion

**Figure 3:**
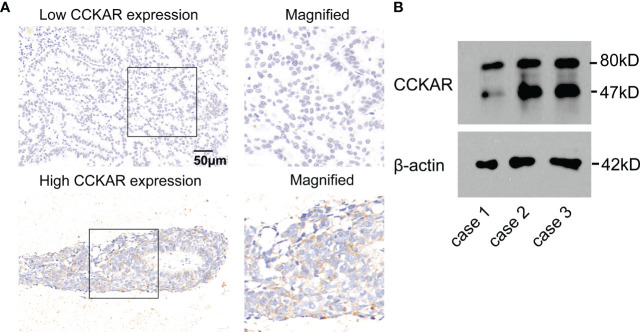
CCKAR expression in BM from NSCLC. **(A)** The expression of CCKAR in BM from NSCLC was detected by IHC. Patients with BM from NSCLC were divided into subsets with low or high CCKAR expression. **(B)** The expression of CCKAR in 3 BM tissues was detected with Western Blotting.

A total of 43 patients with BM underwent surgical resection, and CCKAR expression in BM lesion was assessed with IHC and WB (**Figure 3A, B**). According to the cut-off of CCKAR IHC score in BM, we divided these 43 patients into subgroups with high or low CCKAR expression, accounting for 23.3% (10/43) and 76.7% (33/43) respectively. These results further indicate that CCKAR may be a driving force of BM from NSCLC.

## Discussion

Almost 10% of NSCLC are diagnosed with metastasis synchronously, and 25%-40% acquire BM during the course of NSCLC (19). As to patients with mediastinal lymph node metastasis of lung cancer, up to 68% of patients eventually suffer BM (20). BM may cause severe symptoms such as neurological defects, cognitive impairment, and emotional difficulties which require surgical treatment (21). For patients with BM, the prognosis is extremely poor with a median survival time about only 3-6 months (22). However, the life span of BM patients who receive early treatment is much longer than those whose treatment is not prompt (23, 24). So identifying early and effective biomarkers which can predict BM and prognosis is a severe and important task for BM treatment. Here in our study, we identified CCKAR as a prognostic biomarker of NSCLC, and showed that CCKAR in NSCLC was significantly associated with asynchronous BM. CCKAR was significantly associated with asynchronous BM probability of NSCLC. Our results indicated that patients with high CCKAR should receive more severe surveillance for BM. This is very helpful to select the patients with high risk of BM and guide stricter review and early treatment.

Treatment to patients with BM from NSCLC has not got total consensus (25). The treatments depend on the performance status, the effect of other adjuvant therapy and overall health of the patient (18). Many conflicts exist as to the treatment options and the large-scale and multi-centered cohort study should be conducted. Identifying systemic treatment guidelines based on the molecular pattern of BM is an urgent task for the precision treatment of patients with BM. Biomarkers of BM could not only select patients for appropriate cancer management strategies but also help screen more effective drug targets. Our results not only define CCKAR as a BM indicator of NSCLC, but also imply that CCKAR may be a potential drug target of BM from NSCLC. CCKAR has some available inhibitors such as lintitript, rebamipide and loxiglumide (26), which may be promising inhibitor of NSCLC and BM treatment.

CCK is the most abundant peptides in the gastrointestinal tract and also in the central nervous system, functioning as important hormones as well as neurotransmitters (25, 27). CCKAR is a promising drug target for gastrointestinal and metabolic diseases. In cancer study, the oncogenic role and mechanism of CCKAR are occasionally reported. As a hormone modulating gallbladder motility, CCKAR expression or genetic variation is reported to influence hepatocellular carcinoma and biliary tract cancers (16, 28). Compared with CCKBR, the function of CCKAR in tumorigenesis and tumor progression is much less studied (29). Among all G protein-coupled receptors(GPCRs), CCKAR is unique because it can couple with all G-protein subtypes, including Gαs, Gαi and Gαq. Different downstream signaling of CCKAR regulates distinct functions (30). The underlying mechanism of how CCKAR correlates with poor prognosis of NSCLC is not involved in our study because of the complex signaling network downstream of CCKAR. We wish that more evidence on the important clinical significance of CCKAR could provide more fundamental research of how CCKAR is related with poor prognosis of cancer.

In conclusion, we assessed the expression of CCKAR in NSCLC and BM from NSCLC, and analyzed the correlation between CCKAR expression and clinicopathological factors including BM. We identified CCKAR as a prognostic biomarker of NSCLC. CCKAR expression in NSCLC is positively associated with asynchronous BM, and also is a risk factor of asynchronous BM from NSCLC. Our results indicate that patients with high CCKAR in NSCLC should receive more severe surveillance for BM, which is very helpful to select high-risk patients for individual and early treatment.

## Data availability statement

The original contributions presented in the study are included in the article/supplementary material. Further inquiries can be directed to the corresponding author.

## Ethics statement

The study was approved by the Ethics Committee of the First and Second Affiliated Hospital of Shandong First Medical University. The patients/participants provided their written informed consent to participate in this study.

## Author contributions

NL, SS, ZL, TW, CZ and TX established the cohort and collected the clinical information, NL performed the experiments and analyzed the data. TX wrote the paper. All authors contributed to the article and approved the submitted version.
